# Quantification of collagen fiber properties in alcoholic liver fibrosis using polarization-resolved second harmonic generation microscopy

**DOI:** 10.1038/s41598-023-48887-8

**Published:** 2023-12-13

**Authors:** Saya Matsuzaki, Eiji Hase, Hiroki Takanari, Yuri Hayashi, Yusaku Hayashi, Haruto Oshikata, Takeo Minamikawa, Satoko Kimura, Mayuko Ichimura-Shimizu, Takeshi Yasui, Masafumi Harada, Koichi Tsuneyama

**Affiliations:** 1 Department of Radiology, Institute of Biomedical Sciences, Tokushima University Graduate School of Medicine, Tokushima, Japan; 2 Division of Interdisciplinary Research for Medicine and Photonics, Institute of Post-LED Photonics, Tokushima University, Tokushima, Japan; 3 Department of Legal Medicine, Nihon University School of Medicine, Tokyo, Japan; 4 Tokushima University Faculty of Medicine, Tokushima, Japan; 5 Tokyo Medical Examiner’s Office, Tokyo, Japan; 6 Department of Pathology and Laboratory Medicine, Institute of Biomedical Sciences, Tokushima University Graduate School of Medicine, 3-18-15, Kuramoto, Tokushima, 770-8503 Japan; 7 Division of Next-Generation Photonics, Institute of Post-LED Photonics, Tokushima University, Tokushima, Japan

**Keywords:** Medical research, Pathogenesis

## Abstract

Liver fibrosis is assessed mainly by conventional staining or second harmonic generation (SHG) microscopy, which can only provide collagen content in fibrotic area. We propose to use polarization-resolved SHG (PR-SHG) microscopy to quantify liver fibrosis in terms of collagen fiber orientation and crystallization. Liver samples obtained from autopsy cases with fibrosis stage of F0–F4 were evaluated with an SHG microscope, and 12 consecutive PR-SHG images were acquired while changing the polarization azimuth angle of the irradiated laser from 0° to 165° in 15° increments using polarizer. The fiber orientation angle (*φ*) and degree (*ρ*) of collagen were estimated from the images. The SHG-positive area increased as the fibrosis stage progressed, which was well consistent with Sirius Red staining. The value of *φ* was random regardless of fibrosis stage. The mean value of *ρ* (*ρ*-mean), which represents collagen fiber crystallinity, varied more as fibrosis progressed to stage F3, and converged to a significantly higher value in F4 than in other stages. Spatial dispersion of *ρ* (*ρ*-entropy) also showed increased variation in the stage F3 and decreased variation in the stage F4. It was shown that PR-SHG could provide new information on the properties of collagen fibers in human liver fibrosis.

## Introduction

When external stress or damage is applied, local inflammation occurs in tissues. Damaged tissue is removed by the inflammatory process, and fibrous tissue, mainly extracellular matrix such as collagen fibers, is formed to fill the space to maintain tissue shape and homeostasis. The excessive production and accumulation of collagen fibers beyond the physiological range is called fibrosis. In the liver, fibrosis is caused by chronic inflammation due to alcohol, viral hepatitis, and autoimmune hepatitis. In recent years, chronic inflammation and fibrosis of the liver caused by excessive fat accumulation in hepatocytes, especially in well-nourished industrialized countries, has become a social and medical problem, and this condition has attracted attention as nonalcoholic fatty liver disease (NAFLD) or nonalcoholic steatohepatitis (NASH)^1^. In the liver, the clinical outcomes of the patients such as carcinogenesis and liver failure have been reported to correlate with fibrosis stage and quantified collagen content^2^. Therefore, many basic and clinical studies have been performed to elucidate the mechanism of lever fibrosis. Previously, fibrosis was thought to only progress irreversibly. Following recent advances in antiviral therapy, it has become clear that there is a bidirectional outcome of progression and resolution, depending on the balance between collagen fiber production and degradation, and that reducing the degree of fibrosis reduces the risk of carcinogenesis^3^. However, the risk of carcinogenesis remains even without fibrosis^4^, suggesting that there are residual risks other than the amount of fibrosis, such as the characteristics of fibrosis.

Liver fibrosis has traditionally been assessed by histological methods such as Azan staining and Sirius Red staining. Liver fibrosis is pathologically categorized into five stages from F0 to F4 according to the new Inuyama classification or METAVIR score. The evaluation is not classified by a precisely quantified serial coefficient, but by a subjective staircase score based on the approximate percentage of fibrotic areas in the pathological specimen. For more objective evaluation, some attempts have been made to quantify the amount of fibrosis on images. However, it is still difficult to automatically distinguish between areas of physiological and pathological fibrosis, and this technique has not yet been put to practical use. It has also been noted that the nature of fibrosis varies according to the causative disease. The starting point and the pattern of fibrosis differ depending on the primary disease, such as viral hepatitis and alcoholic liver disease (ALD) or NASH. To understand the pathophysiology of fibrosis progression and regression, to accurately evaluate the therapeutic efficacy of antifibrotic agents, and to clarify the relationship between fibrosis and carcinogenesis for accurate assessment of the risk of carcinogenesis, it is essential not only to quantitatively assess the amount and area of fibrosis but also to accurately characterize the collagen fibers involved in fibrosis.

Second harmonic generation (SHG) microscopy has attracted attention as a new label-free method for evaluating fibrous tissue since early 2000s. SHG is a nonlinear optical phenomenon that generates light with a frequency twice that of the irradiated light (i.e., half the wavelength) when a hyperpolarizable non-centrosymmetric material is irradiated with intense pulsed light. In biological samples, collagen generates strong SHG light and is used for label-free observation of fibrotic tissue in skin, tendons, ligaments, and various other tissues^5–7^. Gailhouste et al. showed that SHG is useful in the assessment and scoring of liver fibrosis in hepatitis^8^. It has also been shown that a combination of SHG and two-photon excitation autofluorescence can be useful in assessing and scoring liver fibrosis due to hepatitis and NAFLD^9^. Since SHG per se can only assess the amount of fibrosis, more recently the concept of polarization-resolved SHG (PR-SHG) has been proposed. PR-SHG is based on the principle that pulsed laser irradiation with linear polarization parallel to the fiber orientation produces strong SHG light, whereas laser irradiation with orthogonal linear polarization produces weak SHG light^10^. PR-SHG can obtain new information on collagen fiber orientation as well as collagen content^11–13^. Although the previous studies have proved the efficacy of PR-SHG in analysis of collagen fibers in vitro and in animal models of liver fibrosis, the usefulness of PR-SHG in human liver tissue has not yet been verified. In the present study, we aimed to quantitatively evaluate the properties of collagen fibers in patients with ALD in terms of the orientation and crystallization using PR-SHG microscopy, and verified its potential for future clinical application.

## Results

### Correlation of SHG images and Sirius Red staining

**Figure 1:**
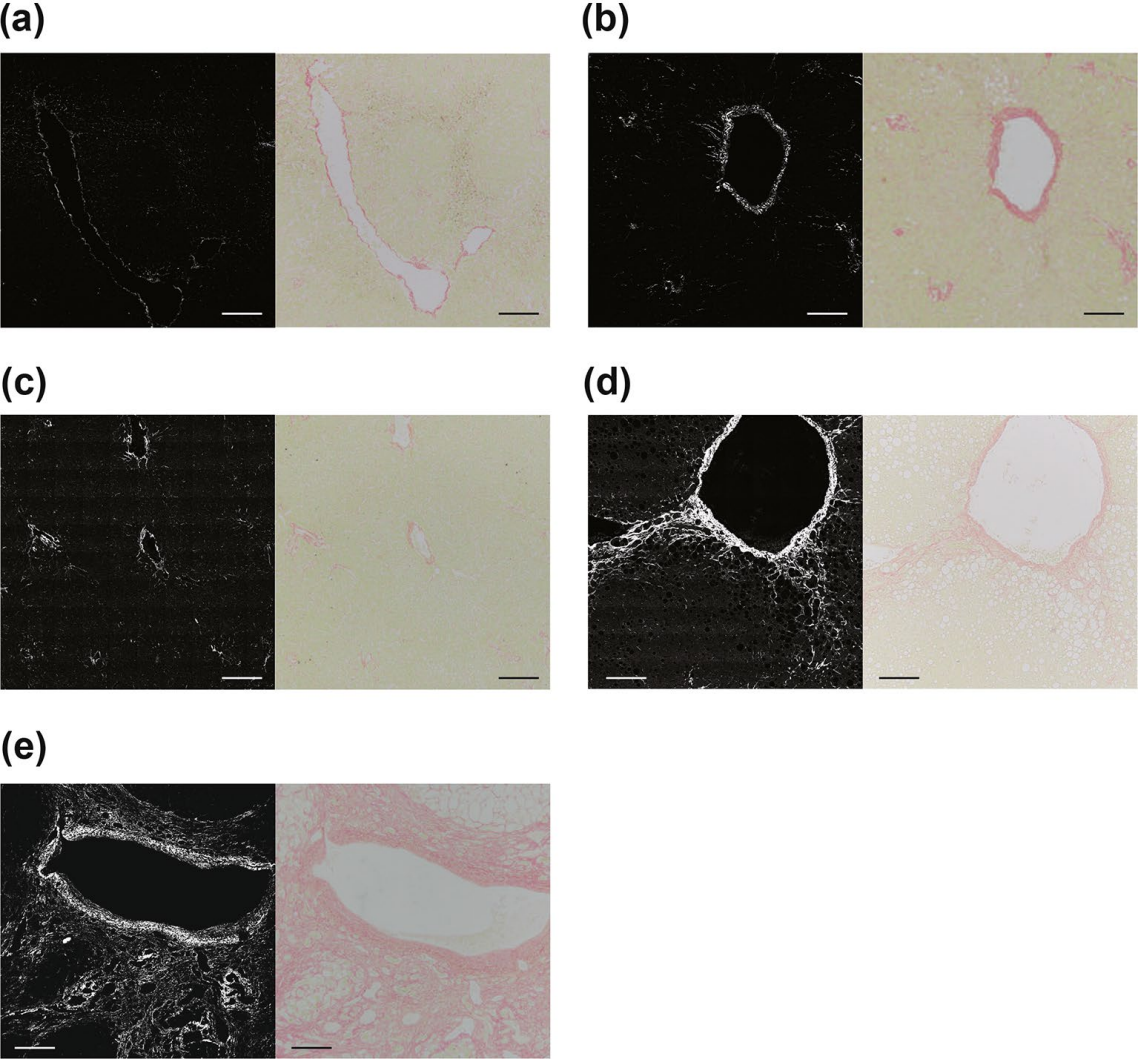
Representative SHG and Sirius Red-stained images in each stage of fibrosis. Of two consecutive liver sections, one was imaged with SHG microscopy (left) and the other with Sirius Red staining (right). Fibrosis was compared in the same region around a central vein. Representative photographs are shown for each stage of fibrosis from F0 to F4 (corresponding to panels (**a**) through (**e**)). The scale bars represent 200 µm.

**Figure 2:**
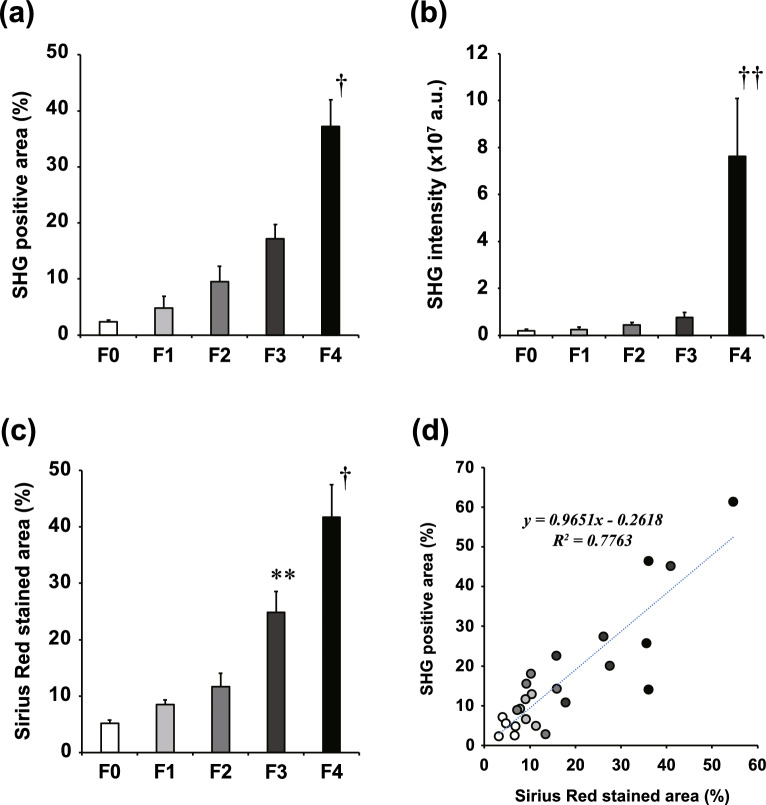
Summarized data and correlation of SHG and Sirius Red-stained images. (**a**) Percentage of SHG-positive area at each fibrosis stage. (**b**) Total SHG intensity within the observation area at each fibrosis stage. (**c**) Percentage of Sirius Red-stained area at each fibrosis stage. (**d**) Scatter plot of SHG-positive area *vs.* Sirius Red-stained area. ^†^*p* < 0.01 vs. F0, F1, F2, and F3. ^††^*p* < 0.05 vs. F2 and F3. **p < 0.05 vs. F0, F1, and F2. One-way ANOVA with Tukey’s post-hoc test.

We first compared the appearances of fibrotic regions indicated by Sirius Red staining and by wide-area SHG imaging. Since this study focused on ALD, the comparison focused on fibrosis around the central vein, which is a characteristic pathological finding of ALD. Figure 1 shows representative images of SHG microscopy and Sirius Red staining of the same region around a central vein in consecutive serial sections of the sample. The SHG-positive areas were highly consistent with positive areas of Sirius Red staining. As shown in Fig. 2a, the percentage of SHG-positive pixels to total pixels in the wide-area SHG image increased as the fibrosis stage progressed, with significantly large SHG-positive areas in F4 stage than in other stages. The total SHG light intensity also increased as the fibrosis stage progressed, and was markedly increased in stage F4 (Fig. 2b). The percentage of Sirius Red-stained area to the total area of the specimen increased with progression of fibrosis stage (Fig. 2c), showing the same trend as for SHG-positive area (Fig. 2a). There was a good correlation of the percentage of SHG-positive area with the percentage of Sirius Red-stained area in the same sample (correlation coefficient [*R*^2^] = 0.7763) (Fig. 2d).

### Quantitative assessment of collagen fiber orientation

**Figure 3:**
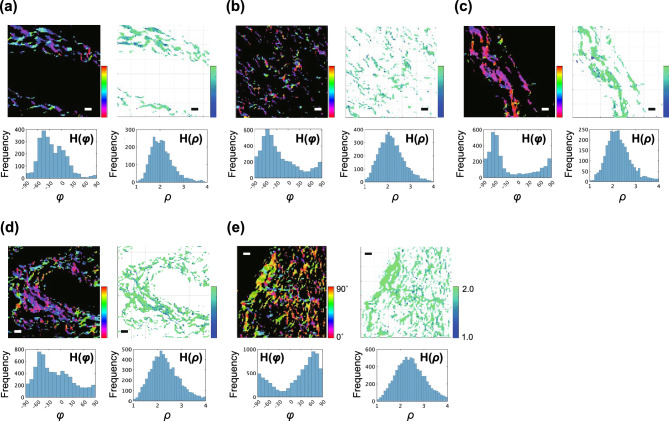
Representative PR-SHG images and histograms of φ and ρ. Representative PR-SHG images of *φ* (upper left) and *ρ* (upper right) and histograms for *φ* (bottom left) and *ρ* (bottom right) based on each image obtained from samples of each stage of fibrosis from F0 to F4 (corresponding to panels (**a**) to (**e**)). The scale bars represent 10 µm.

Collagen fiber orientation was evaluated by creating color maps and histograms of orientation angle (*φ*) and degree (*ρ*) (H(*φ*) and H(*ρ*), respectively) as shown in Fig. 3. The value of *φ*, which indicates the orientation angle of collagen fibers in the observed area, showed wide variation from sample to sample and image to image even within the same fibrosis stage, and we could not find any apparent tendency according to fibrosis stages. On the other hand, the value of *ρ*, which represents the crystallization of collagen fibers in the observed area, showed that the peak shifted to higher values as the fibrosis progressed.

**Figure 4:**
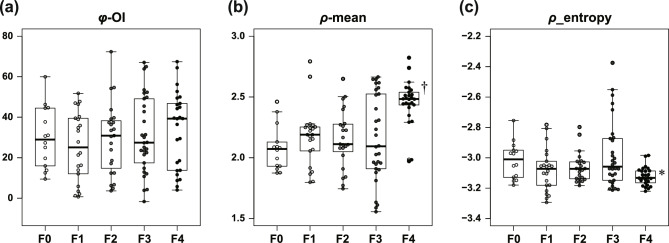
Summarized data of φ-OI, ρ-mean, and ρ-entropy. The data of φ-OI (**a**), ρ-mean (**b**), and ρ-entropy (**c**) compiled into box-and-whisker diagrams are superimposed on a plot of actual data from F0 to F4. The boxes indicate the inter-quartile range (IQR) and the bars above and below the boxes indicate the range between maximum and minimum values. ^†^*p* < 0.01 vs. F0, F1, F2, and F3. *p < 0.01 vs. F3. One-way ANOVA with Tukey’s post-hoc test.

**Figure 5:**
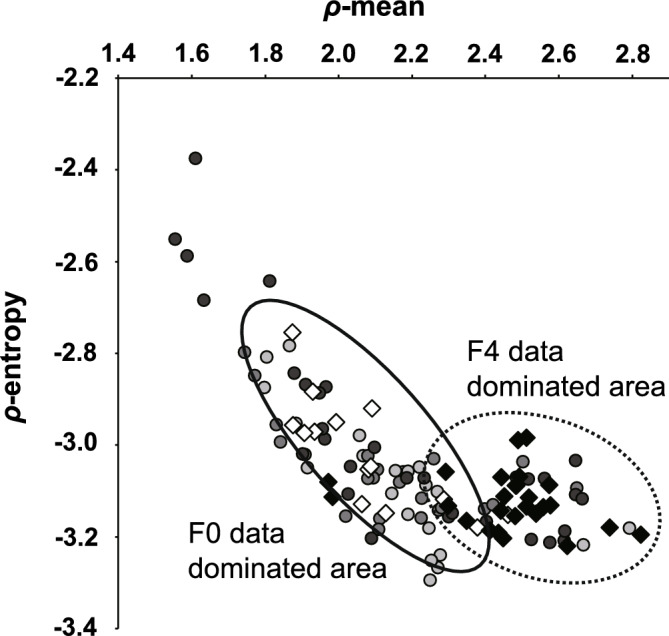
Scatterplot showing the distribution of ρ-entropy relative to ρ-mean. White diamonds indicate F0, gray circles indicate F1–F3 (light, medium, and dark grey, respectively), and black diamonds indicate F4 data. The solid and dotted ovals indicate areas where the scatter data accumulates in stages F0 and F4, respectively.

Figure 4 summarizes the *φ* and *ρ* data. The orientation index of *φ* (*φ*-OI) as a measure of variation in collagen fiber orientation revealed no statistical difference by fibrosis stage (Fig. 4a). The mean value of *ρ* (*ρ*-mean), which represents the degree of collagen fiber crystallinity, showed a very characteristic change. In stage F0, *ρ*-mean was approximately 2.1 and the inter-quartile range (IQR) was 0.189, showing little variation. As fibrosis stage progressed from F1 to F2, the average and median values of *ρ*-mean did not change significantly, whereas the IQR increased slightly to around 0.20. At stage F3, there was little change in the average and median values but the IQR was 0.604, indicating large variation in the data. At stage F4, *ρ*-mean was 2.5, which was significantly higher than at any other stage, and the IQR was 0.097, showing less variation (Fig. 4b). A similar trend was observed for *ρ*-entropy, which represents spatial heterogeneity of collagen fiber crystallinity in the observed area. The IQR of *ρ*-entropy was higher at stage F3 (0.264) than stages F0–F2, although there was no change in the mean or median values. At stage F4, the average value of *ρ*-entropy (− 3.12) was significantly lower and the IQR (0.075) showed less variation compared with other stages. We further evaluated the relationship between *ρ*-mean and *ρ*-entropy of each of the two samples. When *ρ*-entropy was plotted against *ρ*-mean, all data were linearly distributed from the upper left (i.e., the region of low *ρ*-mean and high *ρ*-entropy) to the lower right (i.e., the region of high *ρ*-mean and low *ρ*-entropy) (Fig. 5). Among them, data from F0 samples were concentrated in the upper left of the scatter plot (solid oval), whereas data from F4 samples were concentrated in the lower right of the scatter plot (dotted oval). The data for the F1 to F3 stages were widely distributed among these data.

## Discussion

In the present study, we assessed fibrosis due to alcoholic liver injury in human postmortem tissue using SHG microscopy. The major findings of the study were as follows. (1) SHG microscopy could accurately detect liver fibrosis, and there was good correlation of SHG images with fibrosis stage based on Sirius Red staining. (2) We have confirmed for the first time that PR-SHG microscopy could estimate the orientation angle (*φ*) and degree (*ρ*) of collagen fibers in human liver tissue. (3) We further evaluated collagen fiber orientation in terms of *φ*-OI, *ρ*-mean, and *ρ*-entropy in human liver tissue. (4) *φ*-OI showed no obvious correlation with stage of fibrosis, whereas rho-mean and rho-entropy showed data dispersion in the process of liver tissue inflammation caused by alcohol and data convergence in cirrhosis.

In recent years, the development of new imaging techniques such as shear-wave elastography (SWE), magnetic resonance elastography (MRE), and transient elastography (FibroScan^®^) has been actively pursued for quantitative evaluation of liver fibrosis in a noninvasive manner^14^, which is becoming increasingly important as the number of patients with NAFLD continues to increase rapidly. Following the development and use of anti-fibrotic agents in clinical medicine^15,16^, it is also becoming increasingly necessary to distinguish between drug-responsive and drug-resistant fibrosis to clearly define the treatment criteria. SWE and MRE enable noninvasive assessment of fat accumulation and fibrosis in the liver, and their diagnostic value has been increasing in recent years due to enhanced spatial resolution. The FibroScan^®^ is commonly used for noninvasive monitoring of the progression and regression of liver fibrosis^17^. SWE has also been shown to accurately reflect changes in liver stiffness induced by antiviral treatment in viral hepatitis^18^. MRE can analyze the elastic and viscous components of the liver independently by applying external vibration with different frequencies, which is helpful in distinguishing fibrosis, inflammation, and congestion^19^. These new methods have the advantage of noninvasive diagnosis, however, confounding factors and spatial resolution still remain problems^20,21^. Electron microscopy is a method to observe collagen fibers in more detail. However, electron microscopy is time-consuming to prepare samples, and it is questionable whether it accurately reflects the clinical phenotypes because of the artifacts during sample preparation. On the other hand, SHG microscopy can be used to observe organs immediately after removal as long as a thin slice specimen is prepared, and organs can be observed as they are if a microscope with a reflective configuration is prepared. The assessment of liver fibrosis by PR-SHG would enable the evaluation of fibrosis specific to collagen fibers at the microscopic level and have potential to bridge the gap between macro (such as liver stiffness and serum biomarkers) and micro information (such as collagen fiber properties).

SHG imaging has recently attracted attention as a novel label-free evaluation of fibrotic tissues and collagen fibers. It has been used to visualize and evaluate collagen in soft tissues such as skin, tendons, and ligaments, as well as pathological fibrosis in cancer and liver injury^5–9,22^. We found high consistency between SHG imaging and Sirius Red staining according to fibrosis stage, which reaffirms the potential of SHG as a method for assessing liver fibrosis. However, the information obtained by SHG microscopy is limited to the amount and distribution of collagen and not up to the superiority over conventional staining evaluation. PR-SHG microscopy additionally provided us the indices of collagen fiber orientation angle (*φ*) and degree (*ρ*), which are not available by conventional microscopy or SHG microscopy with normal configuration. Among them, we could not find any statistically significant difference in *φ* corresponding to fibrosis stages in human liver tissue. As *φ* represents fiber orientation, it could be reasonable to use PR-SHG microscopy to assess pathological changes in tissues where fibers are oriented uniformly in a certain direction, for example in tendons and ligaments. On the other hand, in fibrosis of parenchymal organs such as the liver, fibrosis occurs to fill in tissue damaged by inflammation and other factors, so the orientation of collagen fibers is not aligned in a certain direction. This might be the reason why little difference in *φ* depending on fibrosis stage was observed in liver fibrosis in the present study. In contrast, the value of *ρ*, which represents the crystallinity of collagen fibers, showed characteristic changes according to the stage of fibrosis. The data in F4 stage had higher *ρ*-mean and lower *ρ*-entropy than the other stages, suggesting that collagen in F4 fibrosis had heterogenous orientation at fibril level, and also had higher spatial heterogeneity in the organ. In self-assembling collagen gel and in a rat model of liver fibrosis induced by bile duct ligation, the anisotropy of SHG and the ratio of forward to backward scattering of PR-SHG were shown to change, possibly due to an increase in type III collagen relative to type I collagen^23–25^. The previous article also suggested SHG images in breast cancer differed depending on cross-linking between collagen fibers^26^. The morphological organization of collagen within breast cancer samples depends not only on cross-linking but also on its supramolecular symmetry, that can be assessed using PR-SHG microscopy^27^. The changes in *ρ*-mean and *ρ*-entropy that we observe in the study might represent the changes in collagen type or in the cross-linking status between collagen fibers. Thus, it was significant to confirm that such collagen fiber-related data could be obtained from human liver tissue by PR-SHG for the first time, even though from postmortem samples. In the future, the ratio of type I to type III collagen or the cross-links between collagen fibers could be correlated with the value of *ρ*-mean and *ρ*-entropy obtained by PR-SHG to provide a more accurate information of the nature of fibrosis.

Liver fibrosis was previously thought to be a unidirectional process that worsened with inflammation and accumulation of tissue damage and was generally staged by the new Inuyama classification or METAVIR score, which determines the fibrosis stage based on the amount of visually observed fibrotic tissue. Recent basic research has shown that fibrosis in parenchymal organs such as liver could have both progressive and regressive outcomes, depending on the balance of collagen fiber formation and degradation. Furthermore, because the extent and timing of inflammatory cell infiltration into the tissue is not uniform, inflammation in the tissue is spatiotemporally heterogeneous, which would be expected to result in spatiotemporal heterogeneity in the formation and degradation of fibrosis. In contrast, in cases where fibrosis has progressed significantly to cirrhosis, the processes of collagen fiber formation and degradation are inactivated, and fibrosis is considered to be irreversible and to lose its spatial and temporal heterogeneity. Our experimental data could support this pathophysiology of the fibrotic process in the liver. In the present data, the variation in *ρ*-mean and *ρ*-entropy values increased as the fibrosis stage progressed from F1 to F3, and the F4 samples showed less variation in *ρ*-mean and *ρ*-entropy. Especially in the case of F3 samples, it was assumed that there was a large variation in the data due to temporal heterogeneity in the progression of fibrosis as well as sample-to-sample variation. In contrast, stage F4 was dominated by complete and stable fibrosis, resulting in less variation in the data. This speculation was also supported by the finding that total SHG intensity was significantly higher in the stage F4 samples than in the others, as it has been suggested that SHG luminosity is higher in thicker, mature collagen fibers^28,29^. Thus in liver fibrosis that repeatedly progresses and regresses, the PR-SHG might be useful in precisely identifying the pathological state of the patients, and in the future, it is expected to be combined with morphological classifications such as the new Inuyama classification and METAVIR score to enable more detailed classification of liver fibrosis. If such knowledge can be accumulated, it might enable us to predict whether fibrotic regions have a tendency to form and expand or to break down and shrink, whether they still have plasticity or already in irreversible state, and even the responses to anti-fibrotic therapy.

The present study has several limitations. First, it should be noted that the study was validated only in cases of ALD. Pathological conditions that produce liver fibrosis include viral hepatitis and NAFLD or NASH, which has been viewed as a major medical problem in recent years. It should also be noted that this study just focused on cases of ALD used postmortem samples and evaluated only one point for each specimen. However, all the autopsy cases analyzed in this study were untreated patients with respect to alcoholic liver disease, which enabled evaluation of samples in a purely fibrotic state according to stage without any intervention. In addition, since ALD is mainly characterized by fibrosis around the central vein, it was relatively easy to interpret the PR-SHG data by focusing on the area around the central vein for comparison. Nevertheless, it was still difficult to interpret data with large individual differences and spatial heterogeneity among samples and temporal heterogeneity due to the time phase of the pathology, and we have not been able to fully examine how this heterogeneous data reflected the clinical pathology. To more accurately understand the meaning of *ρ*-mean and *ρ*-entropy in the pathogenesis of liver fibrosis, it will be necessary to conduct animal experiments in which liver fibrosis can be observed over time in single individuals, and clinical studies in which patients treated with antifibrotic therapy are followed over time. By integrating the various information obtained from these studies, such as biomarkers, imaging findings such as ultrasound and CT, fibrosis classification based on conventional staining methods such as the new Inuyama classification and METAVIR score, quantitative information on the amount of fibrosis by conventional SHG imaging, and quantitative information on the nature of fibrosis by PR-SHG, it would be possible to classify fibrosis in a new and more detailed way that is more relevant to clinical symptoms and treatment indications.

## Methods

### Human liver samples

Among forensic autopsies performed at the Office of the Tokyo Metropolitan Coroner between 2016 and 2019, we analyzed liver samples obtained from patients whose medical histories confirmed their constant alcohol consumption before death (Table 1). The degree of fibrosis in each case was assessed by Dr. Kimura, the forensic scientist in charge, using formalin-fixed paraffin-embedded (FFPE) specimens of the liver, according to the new Inuyama classification. No personal information was linked to the specimen number, and use of the specimens was limited to histological evaluation. Ethical review was performed and approved by the Tokyo Metropolitan Medical Examiner's Office for these uses (No. 2019-2). All methods were carried out in accordance with relevant guidelines and regulations. This study used only already existing samples for which autopsies have been performed in the past, and all samples was not identifiable to any specific individual. Therefore, we did not obtain informed consent, and the information about the research was made available to all bereaved families by the postings and the website of Tokyo Medical Examiner’s Office (https://www.hokeniryo.metro.tokyo.lg.jp/kansatsu/kenkyuu/index.html), and the opportunity for the families to refuse the research being conducted was ensured.

**Table 1:** Patient backgrounds of autopsy liver samples.

Fibrosis stage	Age	Gender	Cause of death	Lipid deposition
F0	70	Male	Intra-abdominal hemorrhage due to liver cyst rupture	0
	36	Male	Acute coronary syndrome	0
	57	Male	Cardiac hypertrophy	1
	67	Male	Acute coronary syndrome	1
	57 (57.4 ± 6.0)	Male (5/5)	Cardiac hypertrophy	0
F1	58	Male	Cardiac hypertrophy	1
	56	Male	Alcoholic ketoacidosis	3
	50	Male	Ischemic heart failure	2
	82	Male	Hypothermia	1
	47 (58.6 ± 6.2)	Male (5/5)	Acute myocardial infarction	1
F2	52	Female	Alcoholic ketoacidosis	3
	78	Male	Cardiac hypertrophy	1
	66	Male	Acute alcohol intoxication	2
	51	Male	Alcoholic ketoacidosis	2
	33 (56.0 ± 7.6)	Male (4/5)	Ischemic heart failure	2
F3	59	Male	Alcoholic ketoacidosis	3
	51	Male	Cerebral aneurysm rupture	0
	76	Male	Acute myocardial infarction	1
	56	Female	Acute alcohol intoxication	3
	68 (62.0 ± 4.5)	Male (4/5)	Alcoholic ketoacidosis	1
F4	71	Male	Liver cirrhosis	3
	55	Male	Liver cirrhosis	3
	49	Male	Alcoholic ketoacidosis	3
	53 (57.0 ± 4.8)	Male (4/4)	Liver cirrhosis	1

### Sirius Red staining and conventional analysis of fibrosis

Of two consecutive FFPE sections with 5-µm thickness obtained per case, one was stained with Sirius Red for conventional microscopy and the other was used for SHG microscopy without any staining. For Sirius Red staining, specimens were immersed in a 3:100 mixture of 1% Sirius Red (Muto Pure Chemical, Tokyo, Japan) and saturated picric acid (Wangysong Solution A, Muto Pure Chemical) for 10 min after deparaffinization. The specimens were then dehydrated with 100% ethanol and xylene and coverslipped.

For bright field microscopic analysis of Sirius Red-stained specimens, we used a BZ-X700 automated microscopy system (Keyence Corporation, Osaka, Japan) controlled by a software BZ-X Viewer. Briefly, an image of normal resolution (960 × 720 pixels) was acquired through a 4 × objective lens (CFI Plan Apo Lambda 4X, Nikon, Inc., Tokyo, Japan; magnification = 4; numerical aperture (NA) = 0.20; working distance = 20 mm), and multiple images were combined by tiling to create a macroscopic image of the entire specimen. The images were then stored in a tagged image file format and analyzed using BZ-X Analyzer software ver.1.3.0.3. The hybrid cell counting program was applied using bright field mode and region masking with one-color extraction based on RGB color mode. The analyzed area was set around the periphery of the entire sample within a polygonal region of interest (ROI), and color extraction was performed by setting the cursor on the Sirius Red-stained area inside the ROI and adjusting it so that the entire Sirius Red-stained area was masked within a tolerance of 35–45. The automated measurement outputs the area of the entire sample and the area of the Sirius-Red-stained area, and the percentage of the Sirius Red-stained area to the sample area was calculated from these values.

### SHG microscope setup and PR-SHG analysis

To perform SHG imaging and PR-SHG imaging, we used a custom-built laser scanning SHG microscope based on a wavelength-tunable femtosecond optical parametric oscillator (InSight DeepSee, Spectra-Physics, Inc., Tokyo, Japan; tuning range = 680–1300 nm; pulse duration = ~ 110 fs; repetition rate = 80 MHz) and an inverted optical microscope (Ti2-U, Nikon, Inc.). The central wavelength and the laser power at the sample were set to be 800 nm and 10–20 mW, respectively. The focal spot was scanned two-dimensionally onto the sample using a pair of galvanometer mirrors (GMs), a pair of relay lenses, and an objective lens (CFI Plan Apo Lambda 60XC, Nikon, Inc.; magnification = 60; NA = 0.95; working distance = 110–210 μm). Forward-propagated SHG light was collected via the condenser lens (TI-C-LWD, Nikon, Inc.; NA = 0.52) and then separated from the excitation laser light by a dichroic mirror (FF705-Di01, Semrock, Inc., Rochester, NY, the US) and an optical band-pass filter (FBH405-10, Thorlabs, Inc., Newton, NJ, the US). Finally, the SHG light was detected by a photon-counting photomultiplier (H8259-01, Hamamatsu Photonics, K.K., Hamamatsu, Japan) connected to a pulse counter. Using the above setup, SHG images of a 137 × 137 μm region, composed of 128 × 128 pixels, were acquired at a rate of 1 image/s. To further expand the imaging region, we scanned the sample position horizontally or vertically at intervals of 137 μm using a stepping-motor-driven translation stage every time an SHG image was acquired. Finally, a large-area SHG image of size 1.37 × 1.37 mm was obtained, corresponding to 1280 × 1280 pixels, by stitching 100 SHG images together in a matrix of 10 rows and 10 columns.

Groups of PR-SHG images were acquired using an additional polarization control unit based on a half-waveplate (WPH05M-808, Thorlabs, Inc.) and quarter-waveplate (WPQ05M-808, Thorlabs, Inc.) installed before the GMs. The input linear polarization angles were rotated uniformly from 0° to 165° in 15° steps. All linear polarization states had a good polarization extinction ratio (> 100) at the back pupil of the objective lens.

PR-SHG analysis exploits the difference in SHG light intensity in SHG images acquired while rotating the input linear polarization angle. Following the method used in previous studies^12,30–34^, we retrieved the fiber orientation angle *φ* and SHG anisotropic parameter *ρ* in specimens at single-pixel resolution. Briefly, when linearly polarized laser light is incident at angle *α* on collagen fiber, the resultant SHG intensity *I*_*2ω*_ measured without an analyzer in front of the detector can be written as

1 $$ I_{2\omega } = K \left( {\left| {\rho \;cos^{2} (\alpha - \varphi ) + sin^{2} (\alpha - \varphi )} \right|^{2} + \left| {2\;sin(\alpha - \varphi )\;cos(\alpha - \varphi )} \right|^{2} } \right), $$

where *K* is the proportional constant depending on an experimental condition such as laser power, and *φ* denotes the angle of the main axis of the fiber with respect to the *x* (horizontal)-axis on the SHG image. *α* is also defined as the angle between the incident laser polarization and the *x*-axis on the SHG image. Here, we assumed that the collagen fiber lay in the xy plane and that the laser light propagated in the z-direction in Cartesian coordinates xyz for the local frame. In this formalism, we also assumed that the collagen fiber possessed cylindrical symmetry (C_∞_). In addition, Kleinman symmetry can be applied because of the difference between the photon energy of SHG light and the first electronic transition in the collagen molecule^10^. Therefore, the second-order nonlinear optical susceptibility tensor *χ*^(2)^ has only two independent nonvanishing tensorial components: *χ*^(2)^_xxx_ and* χ*^(2)^_xyy_. *ρ* is the ratio of these remaining components (*ρ* = *χ*^(2)^_xxx_/*χ*^(2)^_xyy_), and we considered that this parameter was inversely proportional to the degree of collagen orientation and/or the structural maturity of collagen fiber. To obtain an analytic solution of these values, we applied linear least square (LLS) fitting^34^ to the polarization-dependent SHG images at single-pixel resolution and reconstructed *φ* and* ρ* images of the same pixel size as the SHG image. Due to the fast-fitting algorism in the LLS method, the total image processing time for an image of 128 × 128 pixels was within 1 min.

To obtain quantitative values from the *φ* and *ρ* images, we created histograms of the *φ* and *ρ* values in each image and calculated three parameters, *φ*-OI, *ρ*-mean, and *ρ*-entropy, as previously described^35–37^.

From the *φ* image, we calculated the orientation index (OI), which reflects the percentage of fibers with orientation angle parallel to the main orientation angle (*φ*_*max*_) in the *φ*-histogram (H(*φ*)), defined as

2 $$ \begin{array}{*{20}c} {OI = \left[ {2\frac{{\mathop \smallint \nolimits_{{ - 90^{ \circ } }}^{{90^{ \circ } }} H(\varphi )\cos^{2} (\varphi - \varphi_{max} )d\varphi }}{{\mathop \smallint \nolimits_{{ - 90^{ \circ } }}^{{90^{ \circ } }} H(\varphi )d\varphi }} - 1} \right]100} \\ \end{array} $$

We then calculated the mean *ρ* value of the *ρ* images (*ρ*_mean_) and the statistical entropy (S), which was defined as

3 $$ \begin{array}{*{20}c} {S = - \sum p(\rho )\ln [p(\rho )],} \\ \end{array} $$

where

4 $$ \begin{array}{*{20}c} {p(\rho ) = \frac{H(\rho )}{{\sum H(\rho )}}} \\ \end{array} $$

is the probability of encountering a pixel that has the value *ρ*. Statistical entropy expresses the randomness of the *ρ* value in the *ρ-*histogram (H(*ρ)*), with lower S values corresponding to a more uniform *ρ-*distribution. By definition of the calculation, a lower value of *ρ*-mean indicates a more crystallized fiber, and a lower value of *ρ*-entropy indicates a higher spatial heterogeneity of fiber crystallinity.

### Statistics

The data are expressed as mean ± standard error of the mean. Differences between group averages were tested by a one-way analysis of variance with a post-hoc test (Tukey’s honestly significant difference) and values of *p* < 0.05 were considered significant. Statistical analysis was performed using R software ver.3.6.3^38^.

## Data Availability

Data obtained from the experiments are available from the corresponding author upon reasonable request.
